# Conjunctival Flap Covering Combined with Antiviral and Steroid Therapy for Severe Herpes Simplex Virus Necrotizing Stromal Keratitis

**DOI:** 10.1155/2015/565964

**Published:** 2015-02-15

**Authors:** Hua Gao, Yanni Jia, Suxia Li, Ting Wang, Yaohong Tan, Weiyun Shi

**Affiliations:** ^1^Shandong Eye Hospital, Shandong Eye Institute, Shandong Academy of Medical Sciences, 372 Jingsi Road, Jinan 250021, China; ^2^Bascom Palmer Eye Institute, University of Miami, 1638 NW 10th Avenue, Miami, FL, USA

## Abstract

Herpes simplex virus (HSV) necrotizing stromal keratitis is a common type of herpetic stromal keratitis (HSK). Antiviral medication alone cannot control the disease, and corticosteroid eye drops may aggravate the ulcer and result in corneal perforation. Amniotic membrane transplantation effectively treats superficial corneal ulcer resulting from necrotizing stromal HSK. However, the efficacy of this approach seems to be limited for more serious cases. This study presented the clinical treatment of severe HSV necrotizing stromal keratitis (ulcer depth greater than half of the corneal stroma) by conjunctival flap covering surgery in 25 patients (25 eyes) combined with antivirus and corticosteroid treatment at Shandong Eye Hospital from January 2007 to December 2013. Clinical results showed that the mean best spectacle-corrected visual acuity improved from preoperative 20/333 to postoperative 20/40 (*P* < 0.05). All patients recovered ocular surface stabilization. There was recurrence in two eyes, which was cured with antiviral medication. Conjunctival flap covering combined with antivirus and corticosteroid treatment is effective in treating severe HSV necrotizing stromal keratitis.

## 1. Introduction

Herpetic stromal keratitis (HSK) is one of the most frequent causes of blindness worldwide, and herpes simplex virus (HSV) necrotizing stromal keratitis is a common type of HSK [1]. HSV necrotizing stromal keratitis may result from direct HSV invasion into the corneal stroma and the subsequent severe topical immune response induced by viral antigens, which cannot be managed with antiviral medication alone due to persistent immune damage from viral antigens [2–5]. Notably, adjuvant corticosteroid eye drops may aggravate the corneal ulcer [6]. A corneal transplant surgery performed at this time will increase the incidence of immune rejection and erosion of the graft-host junction due to active inflammation [7]. Therefore, management of necrotizing stromal keratitis has become one of the challenging problems for ophthalmologists. Amniotic membrane transplantation with medical therapy can effectively treat superficial corneal ulcer resulting from necrotizing stromal HSK [8–10]. However, the efficacy of this combined approach seems to be limited for more serious cases of HSV necrotizing stromal keratitis. Herein, we report a series of patients with HSV necrotizing stromal keratitis who had corneal ulceration exceeding fifty percent of the tissue and were treated successfully with conjunctival flap covering and cleansing of the infection.

## 2. Materials and Methods

### 2.1. Patients

This study was approved by the ethics committee of Shandong Eye Institute. Twenty-five patients (25 eyes) with herpes necrotizing stromal keratitis were referred to Shandong Eye Hospital of Shandong Eye Institute from January 2007 to December 2013. Among them, 22 were male, and 3 were female, with an age range of 21 to 72 years (mean ± standard deviation, 54.2 ± 11.8). Diagnostic criteria were based on previous reports [11]. All patients had a history of recurrent episodes of eye hyperemia associated with pain and decreased vision, with one recurrent episode in 3 cases, fewer than 5 recurrent episodes in 8 cases, and more than 5 episodes in 14 cases. All patients received corneal scraping, culture, and laser scanning confocal microscopy (Heidelberg Engineering GmbH) examination to exclude combined purulent infection of bacterial, fungal, or* Acanthamoeba* etiologies. The patient history ranged from 3 to 22 months (mean ± standard deviation, 16.6 ± 5.3).

The ulcers were from 3 to 9 mm in diameter and greater than half of the corneal stroma (excluding Descemet's membrane) in depth in all patients. All patients presented with corneal stromal erosion and edema and corneal infiltration. Among the 25 cases, the focus of infection was located in the pupillary zone in 12 eyes, including 8 eyes with a lesion of 6–9 mm in diameter. Six eyes had combined neovascularization and vessel hyperemia. Seven eyes had anterior chamber hypopyon of approximately 1–3 mm.

These patients underwent systemic and topical antiviral treatment for more than 1 week, but the corneal ulcer tended toward expansion and aggravation. Conjunctival flap transplantation was performed to cover the ulcers according to their size.

### 2.2. Surgical Technique

Written informed consent was obtained from each patient. Conjunctival flap covering was performed as previously described [12], with a few modifications. Necrotic corneal tissue was removed, and a conjunctival flap was created with the conjunctival epithelium side up to fill the ulcer and to cover the defect. The single- or free-pedicle conjunctival flap was made next to the peripheral cornea in 12 eyes, while the double-pedicle conjunctival flap was placed next to the central cornea to ensure sufficient blood supply in 13 eyes. After the graft was sutured to the edge of the ulceration with interrupted 10-0 nylon sutures, a contact lens was used to reduce irritation due to the sutures.

### 2.3. Postoperative Treatment and Medication

Postoperatively, as dictated by the extent of corneal lesion recovery, antiviral medication was applied topically and systemically with adjuvant corticosteroid eye drops [13]. Acyclovir was administered intravenously (5 mg/kg) every 8 hours for the first week after surgery and orally (8 mg/kg) three times a day for the following 3 months. All eyes were also given acyclovir eye drops (Wuhan Wujing Pharmaceutical Co., Wuhan, China) every 2 hours for 2 weeks and tapered to 4 times every day. Tobramycin and dexamethasone eye drops (Alcon, Puurs, Belgium) were used 4 times every day for 1-2 weeks and replaced by 1% fluorometholone eye drops (Santen, Osaka, Japan) 4 times every day for approximately 1-2 months. Medical treatment was adjusted with the alleviation of symptoms. Ganciclovir ophthalmic gel (Hubei Keyi Pharmaceutical Co., Wuhan, China) was used every night.

Fluorescence staining was performed every day, and the sutures were removed at 7 days after surgery. The contact lens was taken out after suture removal and epithelial healing. If the corneal epithelium was not found to be healed with positive fluorescence staining, a new contact lens was used.

### 2.4. Postoperative Evaluation

All patients were examined monthly for at least 12 months. The postoperative examination included uncorrected visual acuity (UCVA), best spectacle-corrected visual acuity (BSCVA), intraocular pressure (IOP), and corneal status (including ulceration, edema, and neovascularization). Fluorescence staining was used to detect corneal epithelial defects, and laser scanning confocal microscopy was performed to determine the sequence of epithelial cells and the extent of local inflammation. Anterior segment optical coherence tomography (AS-OCT) was performed to visualize the corneal tissue cicatrization proximal to the conjunctival flap.

### 2.5. Statistical Analysis

Data were analyzed by SPSS 11.5 software. Student's *t*-test was used to compare vision among patients. *P* < 0.05 was considered to be statistically significant.

## 3. Results

### 3.1. Clinical Examination

**Figure 1:**
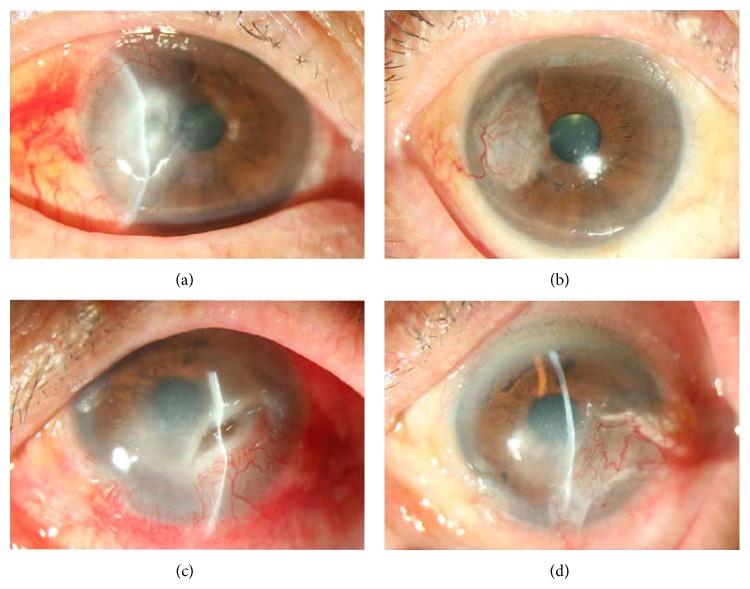
Slit-lamp photographs showing the treatment course of severe herpes simplex virus necrotizing stromal keratitis. (a) Patient 1: the preoperative UCVA was 20/200. The average size of ulcers approximately 4 mm × 5 mm. The depth of the corneal ulcer typically exceeds fifty percent of the entire cornea. Evident corneal necrosis can be seen in the surrounding stromal edema. (b) 12 months after single-pedicle conjunctival flap transplantation, the cornea ulcer was completely cured. The central cornea was clear. The patient had UCVA 20/25 and BSCVA 20/20. (c) Patient 2: the preoperative UCVA was 20/400. Conjunctival hyperemia was observed. The average of the ulcer on the infranasal side of the cornea was about 6 mm × 3 mm. The corneal OCT revealed that the ulcer had nearly reached Descemet's membrane. The surrounding tissue displayed necrosis, infiltration, and edema. (d) 18 months after double-pedicle conjunctival flap transplantation, the corneal ulcer had healed, with the relief of corneal edema, UCVA was 20/63, and BSCVA was 20/32.

All patients were followed up for 12–36 (19.2 ± 5.1) months. All conjunctival flaps were viable without shedding or retropulsion. Corneal ulcers healed within one week postoperatively with negative fluorescence staining (Figure 1). Conjunctival flap ischemia was observed during the earlier period (3–5 days postoperatively) and was followed by hyperemia. Hyperemia decreased gradually when the corneal ulcer healed; the conjunctival flap remained stable and semitransparent. The hypopyon observed in 7 patients disappeared at 1-2 weeks. Corneal stromal edema subsided within 2 weeks postoperatively, with a residual corneal leucoma or macula. The corneal opacity decreased in size during the follow-up, with variable effects on visual acuity. The IOP was within the normal range in all eyes. HSK recurrence was observed in 2 patients (2 eyes) at 6 weeks and 15 months, respectively, without corneal ulcer. Corneal inflammation and recurrence were controlled by topical and systemic antiviral treatment.

### 3.2. Recovery of Visual Acuity

The average preoperative UCVA in 13 eyes with the ulcer lying away from the central 3-mm region of the pupil was 20/63 (range 20/400 to 20/32); the average preoperative BSCVA was 20/40 (range 20/200 to 20/20). The postoperative visual acuity increased by at least one row (maximum, five rows). The UCVA in 12 eyes with the conjunctival flap within the 3-mm region of the central pupil was HM-20/160; the BSCVA was FC-20/125. The percentage of patients whose BSCVA was less than 20/400 decreased from 68.0% (17 eyes) preoperatively to 24.0% (6 eyes) postoperatively. The difference in visual acuity before and after surgery was statistically significant (*P* < 0.05, Table 1).

### 3.3. Confocal Microscopic Examination

Immunocyte infiltration in the necrotizing regions was visualized preoperatively by confocal microscopy. Langerhans cell aggregation and corneal stroma edema were observed in the basal membrane surrounding the necrotizing regions. The shape of the corneal endothelial cells could not be seen clearly.

**Figure 2:**
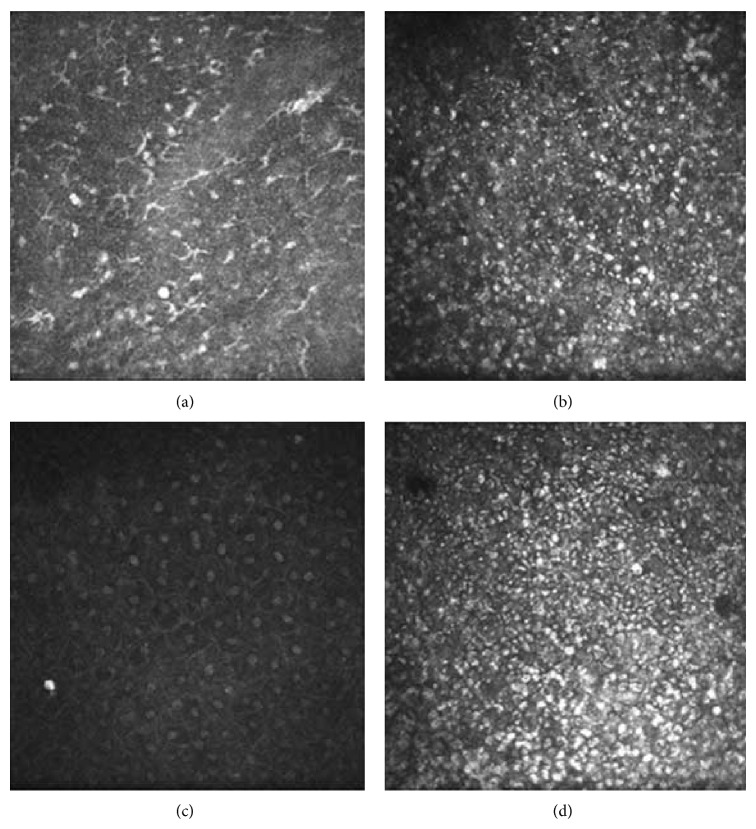
(a) The basal membrane surrounding the necrotizing regions displays abundant Langerhans cells aggregation. (b) Three months after the operation, confocal microscopy indicated that the number of immunocytes had clearly decreased. (c) Normal superficial corneal epithelium cells can be seen in the negative control cornea. (d) 12 months after conjunctival flap transplantation, the conjunctival epithelium cells are replaced by ancipital corneal cells with an epithelial-like appearance, with neovascularization and the proliferation of fibrous connective tissue.

Confocal microscopy revealed that conjunctival epithelial cells covered the ulcer surface. These cells were replaced by ancipital degenerative epithelial cells after 3 months, when neovascularization and Langerhans cell growth were observed, as well as inflammatory cell infiltration (Figure 2).

### 3.4. AS-OCT Examination

**Figure 3:**
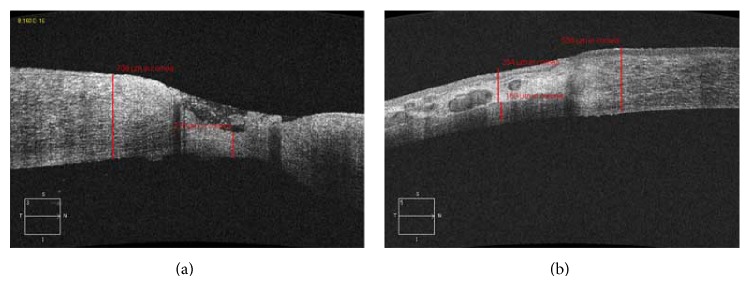
(a) The preoperative AS-OCT showed that the ulcer involved two-thirds of the depth of the corneal stroma. (b) The conjunctival flap healed the cornea tissue, appearing semitransparent and highly reflective.

The conjunctival flap healed the corneal tissue. Postoperatively, the infection focus was thickened, semitransparent, and highly reflective (Figure 3).

## 4. Discussion

The primary clinical manifestation of necrotizing stromal HSK is corneal ulcer and stromal infiltration, which may result from direct HSV invasion into the corneal stroma and the subsequent severe, topical, immune response induced by viral antigens [14, 15]. It was reported that complete virus grains and HSV antigens were detected in the corneal stromal tissue of the HSK patients by immunopathological staining, which improved the pathogenesis of necrotizing stromal keratitis related to HSV infection and the immune response [16–18]. Replicating virus and severe local inflammatory responses may damage the corneal basal membrane and disturb the normal repair process of epithelial cells, which cannot be managed with mere antiviral medication. Corneal perforation frequently results in devastating complications [19]. Topical administration of corticosteroids can suppress the local immune response induced by the virion [8, 13]. However, the presence of an ulcer restricts the use of corticosteroids, which leads to a high risk of corneal melting or perforation. There is an urgent need to determine how to deal with this apparent paradox.

In the current series, a conjunctiva graft provided a healthy substrate, contributing to rapid epithelialization [20]. Using a conjunctival flap for the treatment of chronic corneal ulceration was described by Gundersen [21] in the late 1950s and became a standard surgical procedure. Our group previously found that at least 2-3 weeks were required for superficial ulcer healing after multilayer amniotic membrane transplantation [9], while conjunctival flap transplantation significantly shortened the epithelialization period. The main reasons are as follows: the conjunctival graft comprised conjunctival epithelium cells; the ulcer surface was covered with the conjunctiva immediately after the surgery; the conjunctiva healed rapidly the corneal ulcer (in vivo experiments showing immediate occurrence after conjunctival transplantation) [21, 22]. Khodadoust and Quinter [12] reported that, for patients who underwent conjunctival transplantation, there was usually no epithelial defect after 24 hours. In this study, fluorescence staining showed that it took an average of about 3 days to complete ocular surface epithelialization. Complete ocular surface epithelialization significantly reduced the amount of local necrotic tissue created in response to physical stimuli. The decrease in the number of inflammatory cells was detected by confocal microscopy, which effectively suppressed corneal autolysis and necrosis.

The topical administration of corticosteroids can suppress the local immune response and prevent an adverse reaction to corticosteroids. Although the replicating virus and severe local inflammatory response may lead to direct damage to the cornea, the body's immune response to HSV also leads to local necrosis and ocular inflammation. Multiple dendritic cells surrounding the necrotizing infiltration regions and the endothelium layer were detected preoperatively by confocal microscopy, which indicated that the immune lesions played an important role in the healing process [15]. The ulcer restricted the local administration of corticosteroids. After the corneal surface healed, further use of corticosteroids and antiviral treatment can eliminate the direct immune response and immune lesions.

Although there was a possibility of recurrence after conjunctival flap transplantation [23], the recurrence rate was not very high in the current series. HSV belongs to neurotropic virus. The cornea has a high risk of infection because it contains many nerves. Although the conjunctiva also contains nerves, HSV rarely infects the conjunctiva. In eyes with an HSV infected conjunctiva, the conjunctival ulcer often recovers rapidly [24]. Therefore, conjunctival flap transplantation may be helpful for the ulcer cure and prevention of HSV recurrence.

Conjunctival flap transplantation is useful for patients with deep ulcers [20, 25]. For superficial ulcers, we would recommend multilayer amniotic membrane transplantation. For the patients with deep corneal ulcer, the amniotic membrane may dissolve or shed before the corneal ulcer heals and lead to aggravation of the lesions. The self-conjunctiva facilitates fusion with the cornea and cannot be dissolved. The structure of the conjunctiva tissue includes the epithelium layer, propria lamina, and appendant anadesma tissues. The essential component of the propria lamina is the fibrous layer (fibrous connective tissue and elastic fibers), which can attach rapidly to the corneal stroma and maintain tension to resist the extension.

Because of the cessation of stromal edema, the visual acuity in the majority of cases improved significantly in this study. The average BSCVA was 20/40 in the 13 eyes in which the ulcer was distal to the 3-mm region of the central pupil and FC-20/125 in the 12 eyes in which the conjunctival flap was within the 3-mm region of the central pupil. For the patients with low vision, restoration of a noninflamed ocular surface with conjunctival flap was crucial in enhancing the success of any additional optical corneal transplantation.

Confocal microscopy was used to visualize conjunctival epithelial cells covering the ulcer surface [26]. The conjunctival epithelial cells were replaced by corneal epithelial cells after 6 weeks. Since the conjunctival epithelial cells attach loosely to the basement membrane, while the corneal epithelial cells attach tightly, it is important to protect the epithelium and avoid epithelial cell shedding during the early period after conjunctival flap covering.

We recommend the adjunctive use of antiviral and steroid therapy in the context of conjunctival flap covering for treatment of severe necrotizing stromal keratitis. It helps to promote corneal defect healing, alleviate corneal lesions, and restore useful vision for patients with ulceration lying distal to the 3-mm region of the central pupil. For patients with the ulcer inside the 3-mm region of the central pupil, combined conjunctival flap and medical treatment can rapidly reduce the inflammatory response and provide a stable environment for later keratoplasty to further improve vision.

## Figures and Tables

**Table 1:** The comparison of BSCVA before and after the operation.

Visual acuity	Patients	
	Before	After
<0.05	17	6
0.05–0.3	6	14
0.3–0.8	2	5
	25	25

*χ* ^2^	9.7468	

*P* < 0.05.
